# Linkage between N_2_O emission and functional gene abundance in an intensively managed calcareous fluvo-aquic soil

**DOI:** 10.1038/srep43283

**Published:** 2017-02-24

**Authors:** Liuqing Yang, Xiaojun Zhang, Xiaotang Ju

**Affiliations:** 1 College of Resources and Environmental Sciences, China Agricultural University, 2 Yuanmingyuan West Road, Beijing 100193, China; 2 State Key Laboratory of Microbial Metabolism and School of Life Science and Biotechnology, Shanghai Jiaotong University, 800 Dongchuan Road, Shanghai 200240, China

## Abstract

The linkage between N_2_O emissions and the abundance of nitrifier and denitrifier genes is unclear in the intensively managed calcareous fluvo-aquic soils of the North China Plain. We investigated the abundance of bacterial *amoA* for nitrification and *narG, nirS, nirK*, and *nosZ* for denitrification by *in situ* soil sampling to determine how the abundance of these genes changes instantly during N fertilization events and is related to high N_2_O emission peaks. We also investigated how long-term incorporated straw and/or manure affect(s) the abundance of these genes based on a seven-year field experiment. The overall results demonstrate that the long-term application of urea-based fertilizer and/or manure significantly enhanced the number of bacterial *amoA* gene copies leading to high N_2_O emission peaks after N fertilizer applications. These peaks contributed greatly to the annual N_2_O emissions in the crop rotation. A significant correlation between annual N_2_O emissions and *narG, nirS*, and *nirK* gene numbers indicates that the abundance of these genes is related to N_2_O emission under conditions for denitrification, thus partly contributing to the annual N_2_O emissions. These findings will help to draw up appropriate measures for mitigation of N_2_O emissions in this ‘hotspot’ region.

Nitrous oxide (N_2_O) is a powerful long-lived greenhouse gas and has a 300-times stronger warming effect than that of carbon dioxide in the troposphere on a 100-yr time horizon^1^. It also reacts with ozone in the stratosphere and became the dominant substance in ozone depletion in the 21st century^2^. Nitrous oxide is emitted from both natural and anthropogenic sources, and agricultural soils amended with chemical nitrogen (N) fertilizers and/or manure are mainly anthropogenic sources, which contribute up to 66% of the current global anthropogenic N_2_O emissions^3^ and approximately 75% of anthropogenic emission in China^4^. This makes agricultural soils an important target in the efforts to mitigate anthropogenic N_2_O emissions both regionally and globally^5^.

A better understanding of the processes, mechanisms and factors controlling N_2_O production and emission is a prerequisite for managing agricultural N_2_O emissions. Much progress has been made in the last five decades^6^. Although abiotic reactions are important N_2_O sources under certain circumstances^7^, biological processes play more important roles through at least four microbially mediated processes, i.e., nitrifier nitrification, nitrifier denitrification, denitrifier denitrification and coupled nitrification-denitrification^8^,^9^,^10^,^11^,^12^ which are enhanced by the application of N fertilizers and/or manure to most agricultural soils. The occurrence of each N_2_O production process and its contribution to the total N_2_O emissions depend on the prevailing soil conditions such as pH, temperature, moisture, oxygen, and microbial available C and N^13^,^14^. The above factors could be classified into three categories, i.e., edaphic conditions, climatic conditions and agricultural management practices^15^. Due to the temporal and spatial variation in these factors, specifically measuring N_2_O fluxes and studying the processes among typical soil-climatic regions are important for the mitigation of agricultural N_2_O emissions locally and can greatly contribute to the global total N_2_O budget^3^.

The North China Plain (NCP), an important agricultural region of China with an area of approximate 35 million hectares, is located in northeast China (32–41°N, 113–120°E) on the alluvial plain of the Yellow River and has a warm-temperate sub-humid climate with cold winters and hot summers^16^. The current agricultural practice is a very intensive double-cropping cereal system with irrigated winter wheat and rain-fed summer maize rotations characterized by the application of large amounts of fertilizer N and irrigation with large amounts of groundwater to obtain relatively high yields^16^. These practices lead to substantial total N_2_O emissions in this region which has become a ‘hotspot’ of national N_2_O emissions with global significance^17^. Our laboratory and field experiments on these intensively managed low-carbon calcareous soils over numerous years have found that high peaks of N_2_O emissions (mainly occurred within the first week after fertilization) were always induced by the application of large amounts of NH_4_^+^- or urea-based fertilizers to the soils, with no strong emissions during other periods; even the high nitrate concentration in the soils contribute only a small proportion of the annual N_2_O emissions^18^. Moreover, a total of 67–88% of the N_2_O is emitted during the summer maize season in the annual N_2_O emissions^18^,^19^,^20^,^21^,^22^,^23^,^24^,^25^,^26^,^27^. The N_2_O emission factors are generally lower than the IPCC (2006)^28^ default value (1%), with 0.10–0.59% on an annual basis, 0.08–0.21% in the winter wheat season, and 0.44–0.59% in the summer maize season^16^,^18^,^20^,^22^,^26^,^27^. We speculated that high ammonia oxidation activity linked to denitrification by heterotrophic denitrifiers or by nitrifiers are the major processes causing the observed instant high N_2_O peaks^18^,^24^,^27^,^29^. In the present study we quantify the relevant functional genes of the soil microbial community to further elucidate the biological mechanism of N_2_O emissions in this important region.

The community composition of both nitrifiers and denitrifiers is controlled by distal and proximal factors^30^. Distal factors are those factors that control the composition and diversity of nitrifying and denitrifying communities over the long term, and the proximal factors are those factors that affect instantaneous nitrification and denitrification rates. The ammonia oxidizing bacteria (AOB) community could be shaped by distal factors such as soil pH, ammonia and oxygen availability through which proximal factors such as application of NH_4_^+^- or urea-based fertilizer and/or manure and temporal soil temperature and moisture affect the nitrification rate^31^,^32^,^33^,^34^. Denitrifier community composition is structured over the long term by distal controls including the temperature and moisture conditions, substrate availability, competition and disturbances. The rate and kinetics of denitrification at any particular moment are controlled by proximal factors such as oxygen, carbon and nitrate availability^35^.

It is still not clear how the abundance of the bacterial *amoA* gene for nitrification and functional genes for denitrification respond instantly to N fertilization, how they relate to high N_2_O emission peaks, or how long-term incorporated straw and/or manure affect these linkages in the intensively managed low-carbon calcareous soils of the NCP. Our hypotheses are as follows. Firstly, under temporally high NH_4_^+^ concentrations the ammonia oxidizing bacteria will actively grow and accumulate high concentrations of NO_2_^−^, leading to anoxic conditions which, in turn, induce denitrification by heterotrophic denitrifiers, and the above chain processes cause the high N_2_O emission peaks after N fertilization. Secondly, long-term incorporated straw and/or manure will increase soil organic carbon (SOC) and total nitrogen (TN) and lead to increased abundance of denitrification functional genes as a distal control and the denitrifiers act under depleted oxygen conditions when rainfall or irrigation occur, thus partly contributing to annual N_2_O emissions.

We have therefore quantified the functional genes in soils that were sampled *in situ* three times and have performed an Illumina-based 16S rRNA gene sequencing analysis in the 2012–2013 winter wheat-summer maize rotation based on a long-term field experiment that began in 2006 at the Shangzhuang Research Station in suburban Beijing. N_2_O emissions and the concentrations of ammonium, nitrite and nitrate were determined at the same time. The field experiment had been running for seven years at the time of soil sampling. We were therefore able to effectively quantify the relatively long-term effects of different fertilization regimes including chemical fertilizer N combined with straw and/or manures on the total bacterial copy numbers (16S rDNA) and the populations of ammonia-oxidizing and heterotrophic denitrifying bacteria.

## Results

### Changes in the soil chemical and biological properties after 7 years

The SOC in N_opt_, CN_opt_ and CM increased by 15.8, 18.5, and 67.1%, and the TN correspondingly increased by 10.1, 15.2 and 52.5%, respectively, compared with the N_0_ treatment. This indicates that all N fertilization treatments tended to increase SOC and TN compared to the N_0_ control but the effect was only statistically significant for the manure treatment (Table 1). The soil C/N ratio and NH_4_^+^ concentration did not change significantly, and the ammonium concentration was very low, approximately 2 mg N kg^−1^, among the four treatments.

The nitrate concentration in the N_opt_, CN_opt_ and CM treatments was 38.5, 36.5 and 26.2 mg N kg^−1^, respectively, approximately 1.5 to 2.6 times higher than in the N_0_ control. The soil potential nitrification rate in N_opt_, CN_opt_ and CM was 3.39, 3.90 and 3.80 times significantly higher than in the N_0_ control, respectively. The soil potential denitrification rate in N_opt_, CN_opt_ and CM was 1.09, 1.80 and 3.12 times higher compared with the N_0_ control; CN_opt_ and CM were significantly higher than in the N_opt_ and N_0_ treatments, respectively; and only the CM treatment was significantly higher than the other three treatments.

The soil pH measured on the sampling date of 16^th^ April was approximately 7.57–7.75 and was not significantly different among the four treatments (Table S3). However, it was significantly lower in the three fertilization treatments compared to the N_0_ control when measured on the sampling dates of 9^th^ August and 14^th^ August, indicating that urea hydrolysis and nitrification would reduce the soil pH shortly in the calcareous soil studied^36^.

The above changes in the C and N status among treatments are likely the most important distal drivers of bacterial community composition, the abundance of nitrifier and denitrifier genes and related to instant generation of N_2_O by proximal drivers.

### Nitrous oxide emission

**Figure 1:**
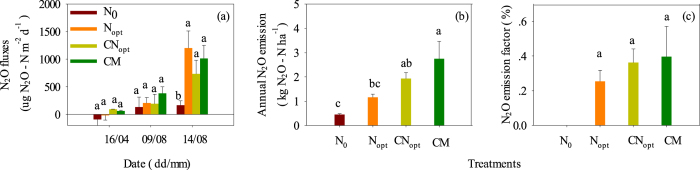
N_2_O fluxes on the sampling dates in 2013 (**a**); N_2_O data in the studied year of the 2012–2013 winter wheat-summer maize rotation (**b**); and N_2_O emission factor (**c**). Different letters in (**a**) indicate significant differences (*P* < 0.05) among treatments on the same sampling date, and different letters in (**b**) and (**c**) indicate significant differences (*P* < 0.05) among treatments in annual base.

**Figure 2:**
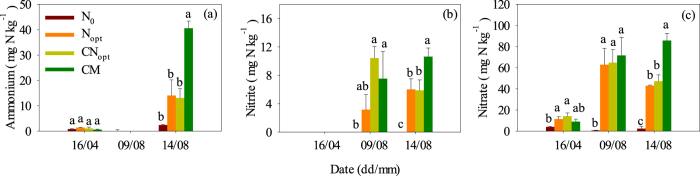
Ammonium, nitrite and nitrate concentrations of different treatments at 0–20 cm soil depth on the sampling dates in 2013. Different letters indicate significant differences (*P* < 0.05) between pairs of treatments.

N_2_O fluxes were very low on 16^th^ April and there were no significant differences among the four treatments (Fig. 1a) during this zero-N fertilization period; there were even negative fluxes in the N_0_ and N_opt_ treatments which may be explained by the low soil temperature (9.6 °C) and moisture content (approximately 11%) in April (Table S3)^20^,^23^. The fluxes appeared to be higher on 9^th^ August than on 16^th^ April in all treatments but were not significantly different (although CM treatment was the highest) due to the rising soil temperature (24–26 °C) and moisture content (approximately 16%) in August, and the higher concentrations of nitrite and nitrate in the soil matrix (Table S3; Fig. 2a,b,c). Surprisingly, there was still 3.2–10.4 mg N kg^−1^ of nitrite in the fertilized soils on 9^th^ August, which is unusual in the soil studied (further explanation in the Discussion section). Although the SOC and TN status changed among the different treatments after 7 years, the N_2_O fluxes did not respond significantly during the periods of zero fertilization in the studied soil.

However, the N_2_O fluxes increased greatly within 7 days during the 10^th^ leaf fertilization of summer maize on the third soil sampling date of 14^th^ August (Fig. 1a). The fluxes in the N fertilization treatments (N_opt_, CN_opt_, CM) were all significantly higher than in the N_0_ control, increasing to 7.0, 4.3 and 6.1-times, respectively, but were not statistically significant among the fertilization treatments, possibly due to the high spatial variation of measured N_2_O fluxes within the field plots. High concentrations of NH_4_^+^, NO_2_^−^ and NO_3_^−^ were detected in all of the fertilization treatments and were significantly higher in the CM treatment. The high NH_4_^+^ concentration in the CM treatment might result mainly from the high N mineralization due to the significantly higher SOC and TN contents in this treatment^37^,^38^ (Table 1). A significantly high NH_4_^+^ concentration in the CM treatment also induced high NO_2_^−^ and NO_3_^−^ concentrations in the soil matrix as a consequence of the typical nitrification chain.

The above results indicate that the high N_2_O emission was mainly derived from the processes of urea hydrolysis to NH_4_^+^ and then oxidation to NO_2_^−^ and finally to NO_3_^−^. These processes were little affected by the distal drivers of the background C and N status but rather by N fertilization events. We will further explain this critical, instant and strong emission period of high N_2_O peaks by bacterial *amoA* abundance in the next section.

The annual N_2_O emissions were in the sequence CM > CN_opt_ > N_opt_ > N_0_ and were significantly higher in the CN_opt_ and CM treatments than in the N_opt_ and N_0_ treatments (Fig. 1b). These emissions were increasing to 2.6, 4.3 and 6.2 times in the N_opt_, CN_opt_, and CM treatments compared to the N_0_ control. This increase corresponded well to the order of the total N applied rate (Table 2) and followed the order of the SOC and TN status among treatments (Table 1). We further calculated the emission factor to investigate the N_2_O emissions per unit of N input in order to determine whether the manure and/or straw N interacted with chemical N to stimulate N_2_O emissions per unit of N input^39^.

The emission factors also increased in the sequence CM > CN_opt_ > N_opt_, but there were no significant differences among these treatments, possibly also due to the high spatial variation of measured N_2_O fluxes in the field plots (Fig. 1c). In our study the annual N_2_O emission factor was 0.20–0.40%, much lower than the IPCC default value of 1%^28^ but was in line with our previous studies^18^,^20^,^23^,^24^,^25^.

### Abundance of the 16S rRNA gene and nitrification and denitrification genes

**Figure 3:**
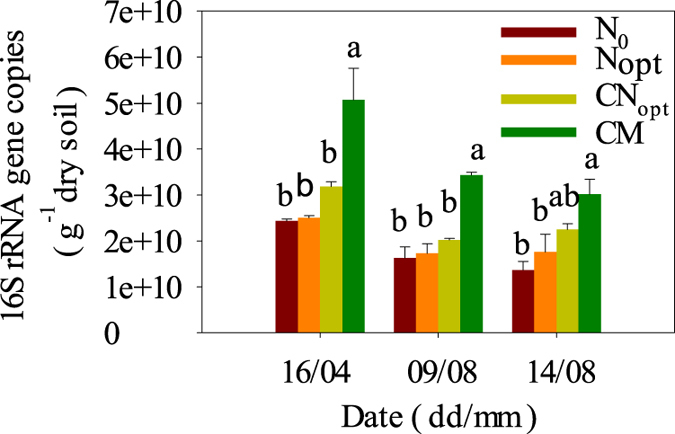
Gene copy numbers of 16S rRNA of different treatments at 0–20 cm soil depth on the sampling dates in 2013. Different letters indicate significant differences (*P* < 0.05) between pairs of treatments.

The order of the 16S rRNA gene copy number was CM > CN_opt_ > N_opt_ > N_0_ on all three sampling dates (Fig. 3), a similar order to SOC, but only significantly higher in the CM treatment compared with the other three treatments. The 16S rRNA gene copy numbers in the CM treatment were 1.6–1.9-, 1.7–2.1- and 1.3–2.2-times larger than in the CN_opt_, N_opt_, and N_0_ treatments on the three sampling dates, indicating that soil with long-term incorporated manure harbored the largest 16S rRNA gene number. Surprisingly, the 16S rRNA gene copies in all four treatments were higher on the 16^th^ April soil sampling date than on 9^th^ August or 14^th^ August, possibly due to the different crops because the first sampling date was winter wheat and the last two sampling dates were summer maize (further explanation in the Discussion section). The soil 16S rRNA gene copy numbers in the CN_opt_ treatment appeared to be 27.2, 16.7 and 27.7% higher than in the N_opt_ treatment on the three sampling dates, indicating that straw return increased the soil 16S rRNA gene copy number but this was not statistically significant.

**Figure 4:**
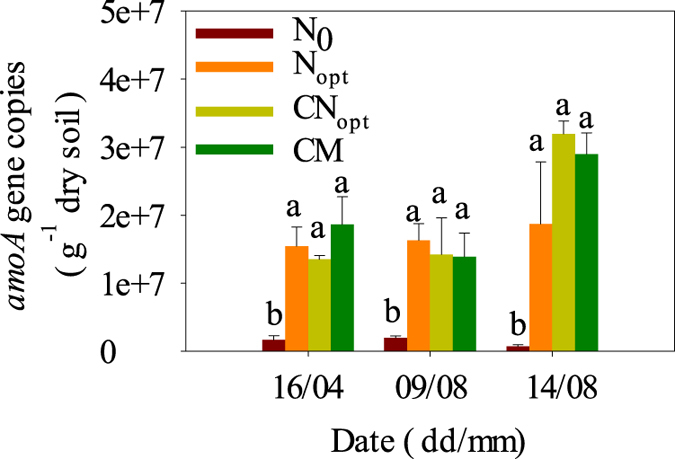
Gene copy numbers of the bacterial ammonia monooxygenase gene (*amoA*) (AOB) of different treatments at 0–20 cm soil depth on the sampling dates in 2013. Different letters indicate significant differences (*P* < 0.05) between pairs of treatments.

The gene copy numbers of bacterial *amoA* in all three fertilization treatments were significantly higher than in the N_0_ control but were not significantly different among fertilization treatments on all three sampling dates (Fig. 4), reflecting the legacy of historical long-term application of urea-based fertilizer and/or manure as distal drivers to regulate nitrification. These numbers were 1.4e + 7 to 1.9e + 7 and 1.4e + 7 to 1.6e + 7 on 16^th^ April and 9^th^ August, respectively. On the third day after the 10^th^ leaf fertilization, the *amoA* gene number of N_opt_, CN_opt_ and CM increased 14.7, 124.5 and 107.6% compared to that before fertilization (on 9^th^ August), respectively, indicating that the 10^th^ leaf fertilization of maize enhanced the *amoA* gene copy number as proximal factor and led to production of high N_2_O emission peaks in this short period (Fig. 1a). This result further confirms our previous conclusion that ammonia oxidation is an engine to generate nitrous oxide in this intensively managed calcareous Fluvo-aquic soil^24^.

**Figure 5:**
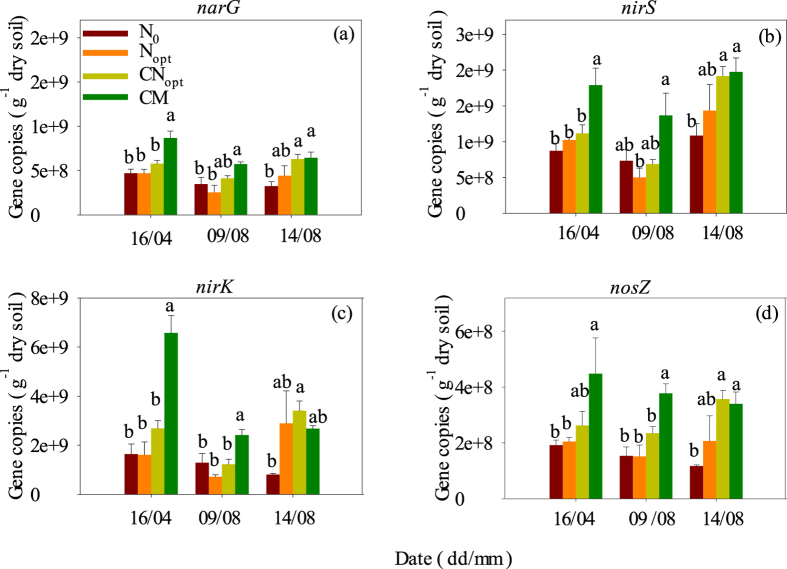
Gene copy numbers of the nitrate reductase gene *narG*, the nitrite reductase genes *nirS* and *nirK* and the N_2_O reductase gene *nosZ* of different treatments at 0–20 cm soil depth on the sampling dates in 2013. Different letters indicate significant differences (*P* < 0.05) between pairs of treatments.

The abundance of the denitrification genes encoding nitrate reductase (*narG*), nitrite reductases (*nirS* and *nirK*) and N_2_O reductase (*nosZ*) followed a similar sequence, namely CM > CN_opt_ > N_opt_ > N_0_, on 16^th^ April and 9^th^ August, but in most cases these genes were significantly higher only in the CM treatment compared with the other three treatments (Fig. 5a,b,c,d), which seems to follow the trend of SOC and TN. The third sampling date on 14^th^ August, after fertilization, likely disturbed this trend, and none of the genes were significantly different among fertilization treatments. These results indicate that the abundance of denitrification genes was regulated by both distal drivers, such as SOC and TN, and proximal drivers, such as N fertilization. The gene copy numbers of *narG, nirS* and *nirK* in different treatments on 16^th^ April and 9^th^ August corresponded well with the daily N_2_O emissions during these two zero fertilization periods and with the annual N_2_O emissions (Fig. 1a,b). The gene copy numbers of *narG, nirS* and *nirK* in different treatments on 14^th^ August seem also to partly correspond with high N_2_O emission after fertilization. Although the *nosZ* gene number was significantly higher in the CM treatment in most cases, the N_2_O emission factor in this treatment was still higher than that of the other treatments, likely due to the effect of the *nosZ* gene being partly offset by increased effects of *narG, nirS* and *nirK* genes.

### Sequencing analysis

**Figure 6:**
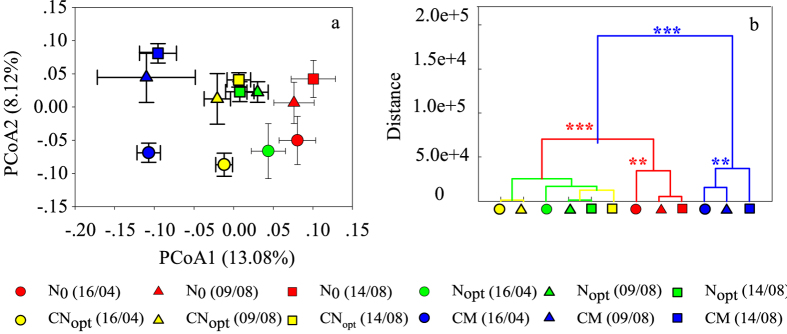
Alterations in the soil microbiota under different treatments at 0–20 cm soil depth on the sampling dates in 2013. (**a**) Principal coordinate analysis (PCoA) score plots based on the Bray-Curtis distance. (**b**) Clustering of soil microbiota based on Mahalanobis distances calculated with a multivariate analysis of variance (MANOVA). Each point represents the mean principal coordinate (PC) score of three replicate soil samples from one treatment at one time point. ***P < 0.001, **P < 0.01.

The PCoA score plots based on the Bray-Curtis distance reveal that fertilization with straw and/or manure changed the structure of the soil microbiota (Fig. 6a) and there were statistically significant differences between the N_0_ control and the fertilization treatments (Fig. 6b), i.e., N_opt_, CN_opt_ and CM were separate from N_0_. Among the fertilization treatments, N_opt_ was separated from the CN_opt_ treatment in the PCoA score plots, but this separation was not statistically significant; CM was significantly separated from the N_opt_ and CN_opt_ treatments (Fig. 6a,b). The bacterial community on 14^th^ August after fertilization was also separated from those on the first two sampling dates. These results further indicate that the soil microbial community was influenced by both distal drivers such as changes in the SOC and TN, and proximal drivers, such as N fertilization application.

### Correlations between N_2_O emission and some soil chemical and biological parameters

The annual N_2_O emissions were significantly correlated with SOC and TN, potential denitrification rate (PDNR), *amoA* gene number, denitrification functional gene numbers (*narG, nirS, nirK, nosZ*) and 16S rRNA gene number by Spearman’s rank correlation analysis (Table 3) but were not significantly correlated with soil potential nitrification rate (PNR), likely due to all of the fertilization treatments having a high soil PNR (Table 1). Thus, soil PNR is not a limiting factor for controlling N_2_O production and emission. All of the other soil properties and abundance of functional genes were significantly correlated with each other except for *amoA* with SOC and TN and PNR with TN, which corresponds well with the above results showing that the *amoA* gene number did not respond to distal control (SOC and TN) but responded strongly to instant fertilization as a proximal control (Fig. 4).

## Discussion

We investigated the bacterial *amoA* for nitrification *in situ* during both the zero-fertilization period and a fertilization event using a long-term field experiment with different fertilization regimes in an intensively managed low-carbon calcareous soil on the NCP. These results show that the long-term application of urea-based fertilizer and/or manure induced and enhanced bacterial *amoA* gene copies, which was related to a strong nitrification process, and consumed O_2_ in the soil matrix then triggered denitrification as shown by our previous study^24^. The N_2_O emission induced by ammonia oxidation is also likely dependent on the heterotrophic respiration rate because incorporation of straw and/or manure aggravated the emissions^23^. This suggests that the ammonia oxidizing bacteria will actively grow under temporally high NH_4_^+^ concentrations, leading to microoxic or anoxic conditions, which in turn induce denitrification by heterotrophic denitrifiers or by nitrifiers, and the above chain processes lead to the high N_2_O emission peaks after N fertilization. The triggering of denitrification was further demonstrated by enhanced *nir*S and *nir*K genes in the current study. Previous studies in the same cropping rotation system showed that these peaks occurring instantly after N fertilization contributed up to 30–70% of the annual N_2_O emissions and were the key periods for controlling N_2_O emissions on the NCP^18^,^20^,^22^,^25^,^27^,^40^. This process may be slowed down using nitrification inhibitors and reducing field N_2_O emissions by up to 77% using DMPP^18^ and 55% using the liquid nitrification inhibitor Piadin^22^. These emissions may be reduced by as much as 80–99% using DMPP or DCD in laboratory experiments^24^,^29^ in calcareous fluvo-aquic soils from the NCP.

Our results provide molecular microbial evidence to illustrate that ammonia oxidation is an engine as the start for generating N_2_O in the study soil, which is why nitrification inhibitors are good for reducing N_2_O emissions^18^,^22^,^24^,^29^ in this soil. The results of the bacterial *amoA* gene agree with other related studies under similar soil and climatic conditions^33^,^37^,^41^,^42^.

It has been reported that AOAs are ubiquitous in soils but do not respond to NH_4_^+^ oxidization or N_2_O production in intensively managed agricultural soils^32^,^43^. Currently, the soils where AOAs have a significant impact on NH_4_^+^ oxidization are mainly acidic^44^. One study conducted in our study region also revealed that *Nitrosospira*-like AOBs were dominant over AOAs in oxidizing NH_4_^+ ^42. Therefore, the contribution of AOAs to N_2_O production is small in our calcareous soil and this is why we did not quantify the *amoA* gene of archaea in our study.

Few studies have investigated the abundance of denitrification genes and their linkage with N_2_O production in our study soil, especially considering the distal and proximal control. We quantified the critical functional genes *narG, nirS, nirK*, and *nosZ* for denitrification and associated their abundances with annual N_2_O emissions. The significant correlations between annual N_2_O emissions and *narG, nirS*, and *nirK* gene numbers show that these genes abundances were related to production of N_2_O under some favorable conditions for denitrification; for example, rainfall or irrigation induce a low oxygen concentration in soil microsites^18^,^19^,^20^,^21^,^22^,^23^,^24^,^25^,^26^,^27^, which may be further supported by the significant correlation between annual N_2_O emissions and SOC and between *narG, nirS*, and *nirK* gene abundances and SOC. These findings further explain our previous results showing that the small pulses occurred when rainfall or irrigation events occurred in the same cropping system, especially during the warm and moist summer^18^,^19^,^20^,^21^,^22^,^23^,^24^,^25^. Therefore, the increased abundance of denitrification genes by long-term incorporated manure can produce N_2_O in our study soil and also partly contribute to the annual N_2_O emissions, which were enhanced by increased SOC and TN. A similar study was conducted using soils sampled in Broadbalk Wheat and the “Broadbalk Wilderness” long-term experiments^45^ but using the incubation technique labeled with KNO_3_ solution to investigate the influence of different long-term fertilization and cultivation treatments on denitrifier communities and to produce N_2_O. The results show that bacteria containing *nirK* were most likely responsible for the increased denitrification potential associated with high SOC and TN, which generally agrees with our findings.

Why was there nitrite accumulation on 9^th^ August (Fig. 2b) when the soil ammonium concentration was quite low (Fig. 2a)? We suspected that it might be due to the reduction of nitrate in more anaerobic microsites together with high levels of dissoluble organic carbon induced by fast root metabolism during the period of strong summer maize growth at higher soil temperatures (approximately 24 °C) and water content (approximately 16%)^46^,^47^. Thus, this nitrite accumulation could contribute to the annual N_2_O emissions. This hypothesis is supported by a recent study that demonstrated the significant contribution of nitrite to N_2_O emissions in maize-cropped soil^29^,^48^.

The explanation for the lower 16S rRNA gene copy number on the two sampling dates of maize (9^th^ and 14^th^ August) than on the first sampling date of wheat (16^th^ April) (Fig. 3) was likely due to competition for resources between crop roots and soil microbes^49^. Summer maize roots might exhaust their resources during fast growth and suppress the growth of microbes, which may result in a lower 16S rRNA gene copy number in the summer maize season. Few studies have reported this phenomenon and it merits further study.

**Figure 7:**
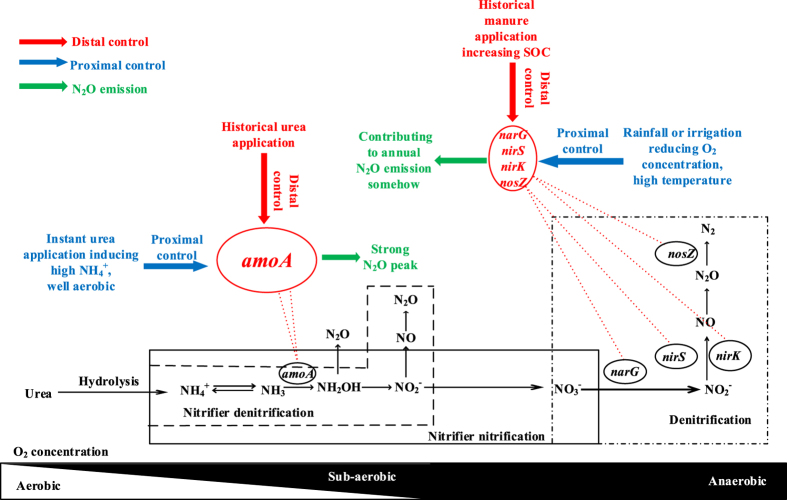
Diagram showing the linkage of N_2_O emissions with functional nitrifier and denitrifier genes controlled by distal and proximal drivers in the intensively managed calcareous fluvo-aquic soil.

Our study highlights the linkage of instant high N_2_O emission peaks with the abundance of the bacterial *amoA* gene for nitrification; annual N_2_O emissions and a small N_2_O pulse after rainfall or irrigation with the abundance of denitrification genes, providing insight into the mechanism of N_2_O production and the factors controlled by distal and proximal drivers in this intensively managed calcareous fluvo-aquic soil (Fig. 7). It is critical to suppress the growth of bacteria containing the *amoA* gene instantly after urea-based fertilization to mitigate N_2_O emission in these strong nitrification soils. We also need to be concerned about the enhanced abundances of denitrification functional genes under favorable conditions.

## Methods

### Site description and soil sampling

The soils on the NCP are derived from alluvial loess transported by the Yellow River and its tributaries and most of the soils are calcareous Fluvisols or Cambisols (FAO Soil Classification System) with a silt texture and relatively uniform profile characterized by high mineralization, high nitrification and low denitrification rates^17^,^38^. The calcareous soils have a pH of 7.5–8.5 and an organic matter content of approximately 10–15 g kg^−1^. Annual cumulative mean temperature for days with temperatures above 10 °C is 4000–5000 °C and the annual precipitation is 500–700 mm with 60–70% of the rainfall occurring during the summer (July–September)^23^. The long-term field experiment began in October 2006 and is located at Shangzhuang Research Station (39°48′ N, 116°28′ E) of China Agricultural University in suburban Beijing. The expression “long-term” in this paper refers to fertilization treatments compared to the no fertilizer control after a period of seven years.

The cropping system was a winter wheat-summer maize rotation. The top 20 cm of the soil profile, sampled at the beginning of the field experiment, had 28% clay, 32% silt, and 40% sand contents and a pH of 8.1 (soil: water ratio 1:2.5). The bulk density was 1.31 g cm^−3^ and the nutrient contents were as follows: SOC 7.1 g kg^−1^, TN 0.8 g kg^−1^, NO_3_-N 24.5 mg kg^−1^, NH_4_-N 1.20 mg kg^−1^, Olsen P 7.8 mg kg^−1^ and available K 76.2 mg kg^−1^. The soil is a typical calcareous fluvo-aquic soil widely distributed across the NCP.

Eight treatments were set up as described in a previous paper^50^. Our current study sampled from four treatments, i.e., N_0_, N_opt_, CN_opt_ and CM, in the 2012–2013 winter wheat-summer maize rotation based on the significant difference of some soil chemical and biological properties at 0–20 cm soil depth sampled before the sowing of winter wheat in 2012 (Table 1). N_0_ is no N application, wheat and maize straw removed; N_opt_ and CN_opt_ are chemical fertilizer N application according to the improved N_min_ (NO_3_^−^-N + NH_4_^+^-N) test, wheat and maize straw removed or returned, respectively; CM is cattle manure supplementary applied N based on the N balance calculation, wheat and maize straw returned. The design is a completely randomized block with three replicates and each plot area is 64 m^2^ (8 × 8 m). Winter wheat was sown at the beginning of October and harvested in the middle of the following June and summer maize was subsequently sown and harvested at the end of September. The chemical N fertilizer used was urea. Straw of both maize and wheat was chopped mechanically into 5–8 cm lengths. Chemical fertilizers and chopped maize straw were incorporated into the soil with tillage at the beginning of October before wheat was sown, and the wheat straw was mulched on the soil surface after the wheat harvest.

In the 2012–2013 winter wheat-summer maize rotation basal fertilizer was applied and winter wheat was sown on 2 October 2012 (Table 2), and winter irrigation (60 mm) was carried out on 17 November 2012. We collected the first soil samples (0–20 cm) at the jointing stage of winter wheat on 16 April 2013 to investigate the effects of long-term treatments on the soil bacterial community composition and the abundance of functional genes. After this sampling, jointing fertilizer was applied on 21 April 2013, followed by 60 mm of irrigation; irrigation (60 mm) was carried out on 14^th^ May 2013 at the heading stage of winter wheat. Winter wheat was harvested on 21^st^ June 2013, summer maize was sown immediately, and the 4^th^ leaf fertilizer of maize was applied on 20^th^ July 2013. The second and third soil samples (0–20 cm) were collected before one day (9^th^ August) and after three days (14^th^ August), respectively, when the 10^th^ leaf fertilization of maize was performed on 11^th^ August 2013 to investigate the effects of N fertilization on N_2_O emissions, the corresponding bacterial community composition and the abundance of functional genes.

Five soil cores were taken from each plot and were mixed to form one composite sample; the samples were stored in an ice box before returning to the laboratory within half a day. The samples were divided into two parts. One part of the fresh soil was used to measure the chemical properties, including ammonium, nitrite, nitrate, water contents and pH, immediately after arriving at the laboratory. The remainder of each sample was stored at −80 °C for subsequent DNA extraction and downstream analysis, including the quantitative real-time PCR (Q-PCR) of the 16 S rRNA gene, functional genes of *amoA* gene of bacterial, nitrate reductase gene (*narG*), nitrite reductase genes (*nirS* and *nirK*), and N_2_O reductase gene (*nosZ*), by high-throughput sequencing of 16S rRNA based on an Illumina platform analysis (Illumina Inc., San Diego, CA).

### N_2_O emission measurements

N_2_O emissions were measured using the closed static chamber method as detailed in Huang *et al*.^23^. They were measured on days of first two soil sampling dates, i.e., on 16^th^ April and 9^th^ August. For third soil sampling day on 14^th^ August, daily measurements were carried out for 7 days after the 10^th^ leaf fertilization on 11^th^ August in order to cover the entire N_2_O peaking period during this N fertilization event. During the whole crop rotation, daily measurements were also carried out for 7 days after each fertilization event and 5 days after each rainfall or irrigation event; for the remaining periods the emissions were measured twice per week and once per week when the soil was frozen^23^.

In the current study N_2_O emissions were calculated daily as μg N_2_O-N m^−2^ d^−1^ on 16^th^ April and 9^th^ August (Fig. 1a), but the N_2_O emissions on 14^th^ August were calculated as the average emissions over 7 days (peak lasting period) after N fertilization in order to reflect the total N_2_O emissions induced by N fertilization. The annual N_2_O emissions (Fig. 1b) were calculated from the sum of measurement days and zero-measurement days (estimated by linear interpolation)^23^. The annual N_2_O emission factor was calculated by subtracting the annual N_2_O emissions of the control treatment from those of the fertilization treatment and then divided by the total N application rate, including straw N and manure N (Fig. 1c), according to IPCC methodology.

### Determination of soil properties

Mineral N was extracted using 1 M KCl solution at a soil:water ratio of 1:5 (w/v) and determined using a continuous flow analyzer (AA3, Seal Analytical, Norderstedt, Germany). Soil nitrite was measured as described by Stevens and Laughlin^51^, the soil nitrification and denitrification potential were determined according to Hart *et al*.^52^ and Tiedje *et al*.^53^ respectively. Details can be found in the Supplementary Information (SI).

### Soil molecular analysis

#### DNA extraction

DNA was extracted from frozen soil using a method based on the CTAB (hexadecyl trimethyl ammonium bromide) method^54^ with some modification, details are described in the SI.

#### Real-time PCR

Quantification of 16 S rRNA genes, and the functional genes *amoA* of ammonia oxidizing bacteria (AOB), *nirS, nirK, narG* and *nosZ* was performed on a Lightcycler 96 system (Roche, Basel, Switzerland) using triplicate samples of diluted DNA (10 ng μl^−1^). Primer pairs of Uni331F/Uni797^55^, amoA-1F/2R^56^, narG-f/r^57^, nirS cd3A/R3cd^58^, nirK1040/FlaCu^59^,^60^, and nosZ-2f/2r^61^ were used for their quantification (Table S1). Each plate included purified plasmid standards and negative controls in triplicate. The amplification efficiency of 16S rRNA, *amoA, narG, nirS, nirK*, and *nosZ* was 87, 85, 86, 78, 85 and 93%, respectively. Much more details can be found in the SI.

#### Illumina-based 16S rRNA gene sequencing

The gene-specific sequences that were used targeted the 16S rRNA gene V3 and V4 regions. The primers that were used for the Amplicon PCR are listed in Table S2. The target fragment was approximately 550 bp. More details can be found in the SI. The 16S rRNA gene sequence information in this study has been submitted to the NCBI Sequence Read Archive (SRA) database under accession number SRP083579.

### Statistical analysis

The data were computed using Microsoft 2010, and the results are reported as the means (±standard error), and the figures were created using SigmaPlot v. 12.5 and Visio 2013. Differences in the soil properties and functional genes were analyzed by one-way analysis of variance and compared by Duncan’s multiple range test at the 5% level using IBM SPSS Statistics 20. We calculated the functional gene numbers considering the effects of treatment and neglected sampling time to assess the significance of correlations between N_2_O emissions and related soil parameters. A non-parametric analysis was used to generate a Spearman’s rank correlation matrix using IBM SPSS Statistics 20. The method of dealing with sequencing data is described in the SI.

## Additional Information

**How to cite this article:** Yang, L. *et al*. Linkage between N_2_O emissions and functional gene abundance in an intensively managed calcareous fluvo-aquic soil. *Sci. Rep.* **7**, 43283; doi: 10.1038/srep43283 (2017).

**Publisher's note:** Springer Nature remains neutral with regard to jurisdictional claims in published maps and institutional affiliations.

## Supplementary Material

Supplementary Information

## Figures and Tables

**Table 1:** Some soil chemical and biological properties (mean ± standard error, n = 3) at 0–20 cm soil depth sampling before the sowing of winter wheat in 2012.

Treatment code	Soil Organic C (g C kg^−1^)	Total N (g N kg^−1^)	C/N Ratio	NH_4_-N (mg N kg^−1^)	NO_3_-N (mg N kg^−1^)	Potential nitrification rate (mg NO_3_-N kg^−1^ d^−1^)	Potential denitrification rate (mg N_2_O-N kg^−1^ d^−1^)
N_0_	7.78 ± 0.92 b^a^	0.99 ± 0.08 b	7.84 ± 0.45 a	1.6 ± 0.9 a	10.6 ± 7.1 b	60.2 ± 17.48 b	1.62 ± 0.10 c
N_opt_	9.01 ± 0.64 b	1.09 ± 0.03 b	8.27 ± 0.89 a	2.0 ± 0.1 a	38.5 ± 4.3 a	204.5 ± 22.65 a	1.76 ± 0.93 c
CN_opt_	9.22 ± 0.95 b	1.14 ± 0.05 b	8.09 ± 0.86 a	1.9 ± 0.2 a	36.5 ± 18.4 a	235.0 ± 45.04 a	2.92 ± 0.73 b
CM	13.00 ± 1.62 a	1.51 ± 0.06 a	8.57 ± 1.05 a	1.6 ± 0.3 a	26.2 ± 5.0 a	228.9 ± 26.56 a	5.06 ± 0.61 a

^a^Within each parameter, different letters indicate significant differences (*P* < 0.05) between pairs of treatments.

**Table 2:** Treatments under a long-term field experiment and nitrogen (N) and carbon (C) rates in the sampling year of the 2012–2013 winter wheat-summer maize rotation.

Treatment code^a^	Nitrogen and straw management	Chemical N fertilization date (dd/mm/yy) and rate (kg N ha^−1^)				Total N input (kg N ha^−1^)	Total C input (T C ha^−1^)
		02/10/2012	21/04/2013	20/07/2013	11/08/2013		
N_0_	No N application, wheat and maize straw removal	0	0	0	0	0	0
N_opt_	Improved N_min_ test, wheat and maize straw removal	75	75	65	65	280	0
CN_opt_	Improved N_min_ test, wheat straw mulching and maize straw return	75	75	65	65	280 (129)^b^	5
CM	Cattle manure with chemical fertilizer N based on N balance calculation, wheat straw mulching and maize straw return	25	73	72	72	242 (157 + 180)^c^	8 (6 + 2)^d^

^a^The same treatment codes are used in the subsequent tables and figures.

^b^The numbers in brackets are straw N.

^c^The numbers in brackets are straw N + manure N.

^d^The numbers in brackets are straw C + manure C.

**Table 3:** Spearman’s rank correlation matrix of annual N_2_O emissions, some soil properties from Table 1, and abundances of the functional genes and 16S rRNA gene (n = 12).

N_2_O^a^	1	1.00										
SOC	2	0.66*	1.00									
TN	3	0.76*	0.84**	1.00								
PNR^b^	4	0.53	0.60*	0.52	1.00							
PDNR^c^	5	0.83**	0.81**	0.76**	0.75**	1.00						
*amoA*	6	0.71*	0.53	0.34	0.73**	0.72**	1.00					
*narG*	7	0.74**	0.78**	0.79**	0.69*	0.90**	0.61*	1.00				
*nirS*	8	0.70**	0.75**	0.82**	0.71*	0.83**	0.59*	0.92**	1.00			
*nirK*	9	0.71**	0.84**	0.69**	0.75**	0.93**	0.77**	0.93**	0.85**	1.00		
*nosZ*	10	0.72**	0.71*	0.59**	0.75**	0.90**	0.82**	0.92**	0.85**	0.97**	1.00	
16S rRNA	11	0.77**	0.80**	0.78**	0.71**	0.93**	0.68*	0.98**	0.92**	0.96**	0.95**	1.00
		1	2	3	4	5	6	7	8	9	10	11

*p < 0.05; **p < 0.01.

^a^Annual N_2_O emission in the 2012–2013 winter wheat-summer maize rotation.

^b^Potential nitrification rate.

^c^Potential denitrification rate.
