# Supplementary material for: Linkage between N2O emission and functional gene abundance in an intensively managed calcareous fluvo-aquic soil
Source: Sci Rep. 2017 Feb 24;7:43283. doi: 10.1038/srep43283 (PMC5324132; doi:10.1038/srep43283)
Supplement: Supplementary Information [file srep43283-s1.doc]

**Supplementary Information**

# Linkage between N_2_O emission and functional gene abundance in an intensively managed calcareous fluvo-aquic soil

Liuqing Yang^a^, Xiaojun Zhang^b,*^, Xiaotang Ju^a*^

^a^ College of Resources and Environmental Sciences, China Agricultural University, 2 Yuanmingyuan West Road, Beijing 100193, China.

^b^ State Key Laboratory of Microbial Metabolism and School of Life Science and Biotechnology, Shanghai Jiaotong University, 800 Dongchuan Road, Shanghai 200240, China

***Corresponding Author: Xiaojun Zhang**

State Key Laboratory of Microbial Metabolism and School of Life Science and Biotechnology, Shanghai Jiaotong University, 800 Dongchuan Road, Shanghai 200240, China. Tel: +86-21-34204878. E-mail: xjzhang68@sjtu.edu.cn

***Corresponding author: Xiaotang Ju**

College of Resources and Environmental Sciences, China Agricultural University, 2 Yuanmingyuan West Road, Beijing 100193, China. Tel: +86-10-62732006. E-mail: juxt@cau.edu.cn

**Material and Methods**

**Soil mineral N measurements:**

Mineral N(including both NH_4_^+^ and NO_3_^-^) was extracted using 1 M KCl solution at a soil:water ratio of 1:5 (w/v) and determined using a continuous flow analyzer (AA3, Seal Analytical, Norderstedt, Germany). Soil nitrite was measured as described by Stevens and Laughlin (1995). Fresh soil (24 g) was shaken in 100 ml of 2 M KCl solution for 10 minutes at pH 8.0, centrifuged for 10 min at 1200 rpm, and filtered. Then, 5 ml of filtered and extracted solution, 15 ml of deionized water and 1 ml of color reagent (sulfanilamide + hydrochloric acid N-1-naphthalenediamine + phosphoric acid) were mixed to form a color that was measured at 543 nm using a UV-VIS spectrophotometer (UV 757 CRYT, Shanghai Precision and Scientific Instrument Co. Ltd, China). The soil water content was determined using the oven-drying method. The SOC and TN were measured using a C/N elemental analyzer (Vario Max CN, Elementar, Hanau, Germany).

The soil nitrification potential was determined according to Hart *et al.*^1^. In brief, 15 g of fresh soil sieved through a 2 mm sieve was placed in a 250 ml conical flask to which 100 ml of nitrification potential fluid (1.5 ml of 0.2 mol L^-1^ KH_2_PO_4_, 3.5 ml of 0.2 mol L^-1^ K_2_HPO_4_, 15 ml of 50 mmol L^-1^ (NH_4_)_2_SO_4_, and 900 ml of deionized water together adjusted to pH 7 at a final volume of 1 L) was added, sealed with parafilm, and shaken (180 rpm). Then, 10 ml of turbid liquid at 2, 8, 14 and 24 h was transferred to centrifuge tubes and spun for 10 min at 5000 rpm, followed by filtration. The NO_3_-N concentration was then measured and nitrification was calculated from the relationship between time and nitrate concentration.

The denitrification potential was measured according to Tiedje *et al.*^2^. Fresh soil (25 g) was placed in a 250 ml conical flask (flask with rubber gasket together with a three-way valve), to which 250 ml of denitrification potential fluid (0.270 g C_4_H_4_Na_2_O_4_·6H_2_O and 0.101 g KNO_3_ dissolved in deionized water, final volume 1 L) was added. The flask was flushed with 99.999% N_2_ for 5 min and sealed, 15-20% C_2_H_2_ was added, the flask was shaken at 180 rpm for 1 h, 5 ml gas samples were collected at 0, 15, 30, 45 and 60 min, the N_2_O content was measured using an Agilent 6820 (Santa Clara, CA), and the denitrification potential was calculated as the N_2_O emissions per unit of soil and time.

**Soil DNA extraction**

DNA was extracted from frozen soil using a method based on the CTAB (hexadecyl trimethyl ammonium bromide) method^3^ with some modification. Frozen soil (0.3 g) and 0.3 g of 0.1 mm glass beads (BioSpec) were added to a 2-ml BeadBeater tube. Extractions were performed by the addition of 0.5 ml of phenol-chloroform-isoamyl alcohol (25:24:1, pH 8) and 0.5 ml of CTAB extraction buffer (equal volume of 10% (w/v) CTAB (Sigma 52365) in 0.7 M NaCl 240 mM phosphate buffer pH 8.0). The samples were treated twice for 45 sec (with 60 s on ice between) at a speed setting of 6.0 m s^-1^ in a Mini – BeadBeater - 8 (BioSpec). The aqueous phase was separated by centrifugation (16,000 × g) for 5 min at 4°C. The aqueous phase was extracted and phenol was completely removed by slowly mixing with an equal volume of chloroform-isoamyl (24:1) and then centrifuging at 16,000 × g for 5 min at 4°C. Total nucleic acids were precipitated from the aqueous phase with 2 volumes of 20% (w/v) PEG (polyethylene glycol 6000, Sigma 81253 in 1.6 M NaCl) on ice for 2 h and centrifuged for 20 min at 16,000 × g and 4°C. The pelleted nucleic acids were washed in ice-cold 70% (v/v) ethanol followed by repeated centrifugation at 16,000 × g for 5 min at 4°C. The supernatant was decanted off and the nucleic acids were air-dried prior to resuspension in100 µl of DNase-free water; 2 µl was used to measure the DNA concentration using the PicoGreen fluorescence method, 2 µl was used to measure the DNA quality on a BioDrop (OD 260/280), 1 µl was used for agarose gel electrophoresis to check the integrity of the DNA, and the remaining DNA was stored at -20°C.

**Real-time PCR**

The 20 µl reaction mixture consisted of 10.0 µl of the iQ^TM^ SYBR® Green Supermix (Bio-Rad, 170-8882), 0.8 µl of each primer (12.5 µM), 6.4 µl of ddH_2_O, and 2 µl (20 ng) of DNA template. A melting curve analysis was performed to confirm PCR product specificity after amplification by measuring fluorescence continuously as the temperature increased from 65 to 95°C and the data analysis was carried out using LightCycler® 96 software (Roche).

Standard curves for real-time PCR assays were developed as follows. Briefly, the 16S rRNA, bacterial *amoA*, *narG*, *nirS*, *nirK* and *nosZ* genes were PCR amplified using the primers 27F/1492R^4^, amoA-1F/2R^5^, narG-1960F/2659R^6^, nirS-cd3A/nirS-R3Cd^7^, nirK-1F/5R^8^ and nosZ-2F/2R^9^, respectively. The PCR products were purified using a PCR cleanup kit (Omega, Cycle-Pure Kit) and cloned into the pGEM-T Easy Vector (Promega). The resulting ligation mix was transformed into DH5α competent cells (TransGen Biotech, Beijing, China) following the manufacturer’s instructions. Plasmids that were used as standards for quantitative analysis were extracted from the correct insert clones of each target gene. The plasmid DNA concentration was determined using the PicoGreen fluorescence method and the copy numbers of the target genes were calculated directly from the concentration of the extracted plasmid DNA. Ten-fold serial dilutions of a known copy number of the plasmid DNA were subjected to real-time PCR in triplicate to generate an external standard curve.

**Illumina-based 16S rRNA gene sequencing**

The five pair primers listed in Table S2 were mixed together and diluted to 5 µM; the target fragment was approximately 550bp. The reaction mixture consisted of 8.5 µl of H_2_O, 12.5 µl of 2 × Phanta Max buffer, 0.5 µl of 10 mM dNTP mix, 1 µl of 5 µM primers (forward and reverse), 0.5 µl of Phanta® Max Super-Fidelity DNA polymerase (P505 – d1, Vazyme, Nanjing, China) and 1 µl of DNA template (10 ng). The reaction conditions were as follows: 95°C for 3 min; 24 cycles of (95°C for 30 s, 55°C for 30 s, and 72°C for 30 s), and 72°C for 8 min. For the Index PCR (attachment of dual indices and Illumina sequencing adapter using the Nextera XT Index Kit), 25 µl reaction mix consisted of 4 µl of H_2_O, 12.5 µl of 2 × Phanta Max buffer, 0.5 µl of 10 mM dNTP mix, 2.5 µl of each N7 and S5 Index primers, 0.5 µl of Phanta ® Max Super-Fidelity DNA polymerase DNA polymerase (P505 – d1, Vazyme, Nanjing, China) and 2.5 µl of purified product of the Amplicon PCR step template DNA. The reaction conditions were the same as for Amplicon PCR except the cycle number was reduced to 8. Other details can be found at http://web.uri.edu/gsc/files/16s-metagenomic-library-prep-guide-15044223-b.pdf.

**Sequencing data analysis**

The method of dealing with sequencing data was described in detail in Zhang *et al.*^10^. All of the analysis was performed based on the QIIME platform^11^. The alpha diversity of each sample was calculated using the observed OTUs and the Shannon index (Fig. S1). Representative sequences for each OTU were built into a phylogenetic tree with Fast Tree and subjected to RDP classifier to determine the phylogeny with a bootstrap cutoff of 80% (RDP database version 2.10). The statistical significance of differences between treatments was assessed by multivariate analysis of variance in MATLAB 2010b (The Math Works Inc., Natick, MA).

**Summary of sequencing on 16S rRNA gene V3-V4 region**

200042 total high-quality reads were obtained for 36 samples. 5557 high-quality reads were obtained for each sample on average. A total of 2980 operational taxonomic units (OTUs) were delineated at a threshold of 97% identity using the Usearch pipline.

**Table S1 Primer set used in the real-time PCR to quantify the 16S rRNA, *amoA*, *nirS*, *nirK*, *narG* and *nosZ*** genes

| Target group | Primer | Sequence (5’-3’) | | | Length of amplicon (bp) | | | Primer concentration  (nM) |
| --- | --- | --- | --- | --- | --- | --- | --- | --- |
| 16S rRNA | Uni331F/Uni797R | tcctacgggaggcagcagt  ggactaccagggtatctaatcctgtt | | | 466 | | | 500 |
|  | 95°C for 180 s; 40 cycles of 95°C for 30 s, 60°C for 30 s, and 72°C for 30 s; and 80°C for 5 s | | | | | | | |
| AOB | amoA-1F/2R | ggggtttctactggtggt  cccctckgsaaagccttcttc | 491 | | | | 500 | |
|  | 95°C for 180 s; 40 cycle of 95°C for 30 s, 60°C for 30 s, and 72°C for 30 s; and 84°C for 5 s | | | | | | | |
| *nirS* | nirS cd3A/R3cd | aacgysaaggaracsgg  gasttcggrtgsgtcttsaygaa | | 425 | | 500 | | |
|  | 95°C for 180 s; 40 cycles of 95°C for 30 s, 58°C for 30 s, and 72°C for 30 s; and 83°C for 5 s | | | | | | | |
| *nirK* | nirK1040/F1aCu | gcctcgatcagrttrtggtt  atcatggtsctgccgcg | | | 473 | | | 500 |
|  | 95°C for 180 s; 40 cycles of 95°C for 30 s, 58°C for 30 s, and 72°C for 30 s; and 80°C for 5 s | | | | | | | |
| *narG* | narG-f/r | tcgccsatyccggcsatgtc  gagttgtaccagtcrgcsgaytcsg | | | 173 | | | 500 |
|  | 95°C for 180 s; 40 cycles of 95°5 for 30 s, 60°0 for 30 s, and 72°C for 30 s; and 80°C for 5 s | | | | | | | |
| *nosZ* | nosZ-2f/2r | cgcracggcaasaaggtsmssgt  cakrtgcaksgcrtggcagaa | | | 267 | | | 500 |
|  | 95°C for 180 s; 40 cycles of 95°C for 30 s, 60°C for 30 s, and 72°C for 30 s; 84°C for 5 s  The data were collected in the last step, the same as for the above genes. | | | | | | | |

**Table S2 Primers used for 16S rRNA sequencing from the V3 to V4 regions**

| Primer | Forward Primer | Reverse Primer |
| --- | --- | --- |
| PFV3V4 | TCGTCGGCAGCGTCAGATGTGTA  TAAGAGACAGCCTACGGGNGGCWGCAG | GTCCGTGGGCTCGGAGATGTGTATAAG  AGACAGGACTACHVGGGTATCTAATCC |
| PFV3V4-1 | TCGTCGGCAGCGTCAGATGTGTAT  AAGAGACAGTCCTACGGGNGGCWGCAG | GTCTCGTGGGCTCGGAGATGTGTATAAGA  GACAGGGACTACHVGGGTATCTAATCC |
| PFV3V4-2 | TCGTCGGCAGCGTCAGATGTGTATA  AGAGACAGCTCCTACGGGNGGCWGCAG | GTCTCGTGGGCTCGGAGATGTGTATAAG  AGACAGTGGACTACHVGGGTATCTAATCC |
| PFV3V4-3 | TCGTCGGCAGCGTCAGATGTGTATA  AGAGACAGACTCCTACGGGNGGCWGCAG | GTCTCGTGGGCTCGGAGATGTGTATAAGA  GACAGATGGACTACHVGGGTATCTAATCC |
| PFV3V4-4 | TCGTCGGCAGCGTCAGATGTGTATA  AGAGACAGGACTCCTACGGGNGGCWGCAG | GTCTCGTGGGCTCGGAGATGTGTATAAGA  GACAGCATGGACTACHVGGGTATCTAATCC |
| PFV3V4-5 | TCGTCGGCAGCGTCAGATGTGTATAA  GAGACAGAGACTCCTACGGGNGGCWGCAG | GTCCGTGGGCTCGGAGATGTGTATAAGAGA  CAGACATGGACTACHVGGGTATCTAATCC |

**Table S3 Some soil parameters (mean ± standard error, n = 3) at 0-20 cm soil depth on the sampling dates in 2013**

| Treatment code | pH | | | | Water content (% ) | | |
| --- | --- | --- | --- | --- | --- | --- | --- |
|  | Date (dd/mm ) | | | | Date (dd/mm ) | | |
|  | 16/04 | 09/08 | | 14/08 | 16/04 | 09/08 | 14/08 |
| N_0_ | 7.57±0.90 a | 7.85±0.03 a | | 8.28±0.10 a | 10.7±0.27 b^a^ | 16.0±0.58 a | 16.9±0.81 a |
| N_opt_ | 7.75±0.04 a | | 7.68±0.04 b | 7.90±0.02 b | 11.4±0.38 ab | 15.2±0.47 a | 15.8±0.77 a |
| CN_opt_ | 7.69±0.44 a | 7.65±0.03 b | | 7.89±0.03 b | 12.2±0.19 a | 15.1±0.47 a | 16.5±0.87 a |
| CM | 7.75±0.03 a | 7.45±0.04 c | | 7.75±0.02 b | 11.8±0.16 a | 15.7±0.71 a | 16.5±0.87 a |

^a^ Different letters indicate significant differences (*P* < 0.05) between pairs of treatments.

Fig. S1 Shannon diversity Index and OTU rarefaction.

**References**

1. Hart, S.C., Stark, J.M., Davidson, E.A.& Firestone, M.K. in Microbiological and Biochemical properties: Nitrogen mineralization, immobilization, and nitrification. Methods of Soil Analysis: Part 2 (eds Bigham, J.M. ) 985-1018 (Madison, 1994).
2. Tiedje, J.M., Sexstone, A.J., Parkin, T.B., Revsbech, N.P.& Shelton, D.R. Anaerobic processes in soil. *Plant Soil* **76**, 197-212 (1984).
3. Griffiths, R. I., Whiteley, A. S., O'Donnell, A. G. & Bailey, M. J. Rapid Method for Coextraction of DNA and RNA from Natural Environments for Analysis of Ribosomal DNA- and rRNA-Based Microbial Community Composition. *Appl.and Environ. Microbiol.* **66**, 5488-5491 (2000).
4. Dees, P.M.& Ghiorse, W.C. Microbial diversity in hot synthetic compost as revealed by PCR-amplified rRNA sequences from cultivated isolates and extracted DNA. *FEMS Microbiol. Ecol.* **35**, 207-216 (2001).
5. Rotthauwe, J.H., Witzel, K.P.& Liesack, W. The ammonia monooxygenase structural gene *amoA* as a functional marker: Molecular fine-scale analysis of natural ammonia-oxidizing populations. *Appl. Environ. Microbiol.* **63**, 4704-4712 (1997).
6. Philippot, L., Piutti, S., Martin-Laurent, F., Hallet, S.& Germon, J.C. Molecular analysis of the nitrate-reducing community from unplanted and maize-planted soils. *Appl. Environ. Microbiol.* **68**, 6121-6128 (2002).
7. Kandeler, E., Deiglmayr, K., Tscherko, D., Bru, D. & Philippot, L. Abundance of *narG*, *nirS, nirK*, and *nosZ* Genes of Denitrifying Bacteria during Primary Successions of a Glacier Foreland. *Appl. Environ. Microbiol.* **72**, 5957-5962 (2006).
8. Braker, C., Fesefeldt, A.& Witzel, K.P. Development of PCR primer systems for amplification of nitrite reductase genes (*nirK* and *nirS*) to detect denitrifying bacteria in environmental samples. *Appl. Environ. Microbiol.* **64**, 3769-3775 (1998).
9. Henry, S., Bru, D., Stres, B., Hallet, S. & Philippot, L. Quantitative Detection of the *nosZ* Gene, Encoding Nitrous Oxide Reductase, and Comparison of the Abundances of 16S rRNA, *narG*, *nirK*, and *nosZ* genes in Soils. *Appl. Environ. Microbiol.* **72**, 5181-5189 (2006).
10. Zhang, Q.P. *et al.* Accelerated dysbiosis of gut microbiota during aggravation of DSS-induced colitis by a butyrate-producing bacterium. *Sci. Rep.*, **6**, 27572 (2016).
11. Caporaso, J.G. *et al.* QIIME allowes analysis of high-throughput community sequencing data. *Nat. Methods* **7**, 335-336 (2010).
